# 

**Published:** 1865-10

**Authors:** 


Volume XXII. Number 10.

### THE CHICAGO

### MEDICAL JOURNAL

DE LASKIE MILLER, M. D.,
EPHRAIM INGALS, M. D.,
Editors and Proprietors.

OCTOBER, 1865.

CHICAGO:

J. C. W. BAILEY, BOOK AND JOB PRINTER, 164 CLARK STREET.

1865.

